# Impact of temperature on dengue and chikungunya transmission by the mosquito *Aedes albopictus*

**DOI:** 10.1038/s41598-022-10977-4

**Published:** 2022-04-28

**Authors:** Aurélien Mercier, Thomas Obadia, Davide Carraretto, Enkelejda Velo, Gaelle Gabiane, Silvia Bino, Marie Vazeille, Giuliano Gasperi, Catherine Dauga, Anna R. Malacrida, Paul Reiter, Anna-Bella Failloux

**Affiliations:** 1 Institut Pasteur, Université Paris Cité, Insects and Infectious Diseases, 75015 Paris, France; 2 Institut Pasteur, Université Paris Cité, Bioinformatics and Biostatistics Hub, 75015 Paris, France; 3 Institut Pasteur, Université Paris Cité, G5 Infectious Disease Epidemiology and Analytics, 75015 Paris, France; 4 Department of Biology and Biotechnology, University of Pavia, Pavia, Italy; 5 Institute of Public Health, Tirana, Albania; 6 Institut Pasteur, Université Paris Cité, Arboviruses and Insect Vectors, 75015 Paris, France; 7 Present Address: INSERM, Univ. Limoges, CHU Limoges, IRD, U1094 Neuroépidémiologie Tropicale, Institut d’Epidémiologie Et de Neurologie Tropicale, GEIST, Limoges, France

**Keywords:** Entomology, Dengue virus, Alphaviruses

## Abstract

The mosquito *Aedes albopictus* is an invasive species first detected in Europe in Albania in 1979, and now established in 28 European countries. Temperature is a limiting factor in mosquito activities and in the transmission of associated arboviruses namely chikungunya (CHIKV) and dengue (DENV). Since 2007, local transmissions of CHIKV and DENV have been reported in mainland Europe, mainly in South Europe. Thus, the critical question is how far north transmission could occur. In this context, the Albanian infestation by *Ae. albopictus* is of interest because the species is present up to 1200 m of altitude; this allows using altitude as a proxy for latitude. Here we show that *Ae. albopictus* can transmit CHIKV at 28 °C as well as 20 °C, however, the transmission of DENV is only observed at 28 °C. We conclude that if temperature is the key environmental factor limiting transmission, then transmission of CHIKV, but not DENV is feasible in much of Europe.

## Introduction

Numerous arboviruses affecting humans are already endemic in continental Europe where they cause sporadic cases during a limited time window fitting with vectors activity; those are West Nile virus, Usutu virus, Tick-borne encephalitis virus, Crimean-Congo hemorrhagic fever virus, and several phleboviruses including Toscana virus^1^. In the last decades, new arboviruses have been introduced in Europe where invasive mosquito species are well established^2^. Among them, five *Aedes* species are described including *Aedes albopictus*. This mosquito has been first detected in Europe, Albania in 1979^3^ and is now present in 28 European countries from Spain to Romania through Germany^4^. Originally from Asia, its high ecological plasticity (i.e. colonizing artificial as well as natural breeding sites) and physiological characteristics (i.e. eggs able to resist to desiccation and freezing) allow this species to spread and successfully establish in both tropical and temperate regions^4^. As expected, *Ae. albopictus*-associated arboviruses were soon after detected in Europe: chikungunya in 2007 in Italy^5^, dengue in 2010 in France^6^ and lastly, Zika in 2019 in France^7^.

Chikungunya hit Italy in 2007 causing more than 200 laboratory-confirmed human cases in Ravenna province from July to September^8^. An East-Central-South-African genotype of chikungunya virus (CHIKV; *Alphavirus*, Togaviridae) was introduced by a traveler coming back from Kerala, India. The virus had the substitution A- > V at the position 226 in the E1 glycoprotein conferring an enhanced replication and transmission by *Ae. albopictus*^9,10^. Besides, dengue was not an unknown disease in Europe. The last documented outbreak happened in 1927–1928 in Greece causing ~ 1 million cases and ~ 1,000 deaths^11^ with *Aedes aegypti* as the main vector. Dengue virus (DENV; *Flavivirus*, Flaviviridae) disappeared from continental Europe following the successful control of *Ae. aegypti* in 1953^12^. However, this species has been detected again around the Mediterranean and Black Sea^13^. While it is described as a bad dengue vector, *Ae. albopictus* can sustain an outbreak in the absence of *Ae. aegypti*; it was involved in the first local cases of dengue in 2010, in France^6^ and Croatia^14^. Since 2010, autochthonous cases of chikungunya and dengue are repeatedly reported in mainland Europe: for dengue, 2013–2015^15–17^, 2018–2020^18,19^ in France, and for chikungunya, 2014^20^, 2017^21^ in France, and 2017 in Italy^22^. These European transmission episodes are usually initiated by imported cases returning from tropical regions during the summer season^23^.

In Europe, the epidemiological landscape of mosquito-borne diseases has drastically changed these last decades mainly as a consequence of the establishment of *Ae. albopictus* in part of Europe in addition to the growing number of people travelling between arbovirus-endemic countries and Europe, and suitable climate conditions conducive to *Ae. albopictus* expansion and proliferation^1^. The first record of *Ae. albopictus* in Europe was reported in Albania in 1979 probably introduced from China^3^. In Albania, the species is commonly found everywhere and even succeeded in reaching high altitudes up to 1200 m^24^. Altitude can be a proxy for temperature which is a critical driver of mosquito activities requiring genetic selection and physiological adaptation of mosquitoes to the cold. Temperature determines mosquito biology, ecology, behavior, and more notably, transmission of arboviruses^25^. In particular, temperature influences significantly adult size^26^ and mosquito females can be larger at high altitudes^27^. Larger females absorb more blood than smaller females^28^, thus potentially increasing the quantity of viral particles ingested by mosquitoes and consequently, the vector competence^29,30^. Alterations in mosquito gene expression and physiology could be observed between small and large mosquitoes^31^. Thus, as mosquitoes may extend their distribution northward, arboviral diseases are expected to emerge in much more of Europe^32^. The critical question is how far north transmission could occur?

In this context, the Albanian *Ae. albopictus* populations are of interest as they colonize high altitudes. These altitude-adapted populations could be a good indicator of latitude-adapted mosquito populations as this invasive species moves northwards into northern Europe. Here we study the effect of temperature on transmission of CHIKV and DENV by *Ae. albopictus* collected at different altitudes in Dajti mountain in Central Albania. We showed that *Ae. albopictus* can transmit CHIKV with similar viral loads in mosquito saliva independently of the incubation temperature tested. In contrast, the transmission of DENV is only observed at 28 °C. Altogether, our study shows that CHIKV transmission can occur at 20 °C (tested as a proxy of high altitudes) increasing the risk of epidemics beyond its current range.

## Results

### *Aedes albopictus* samples collected at high altitudes are genetically different from populations at low altitudes

**Figure 1:**
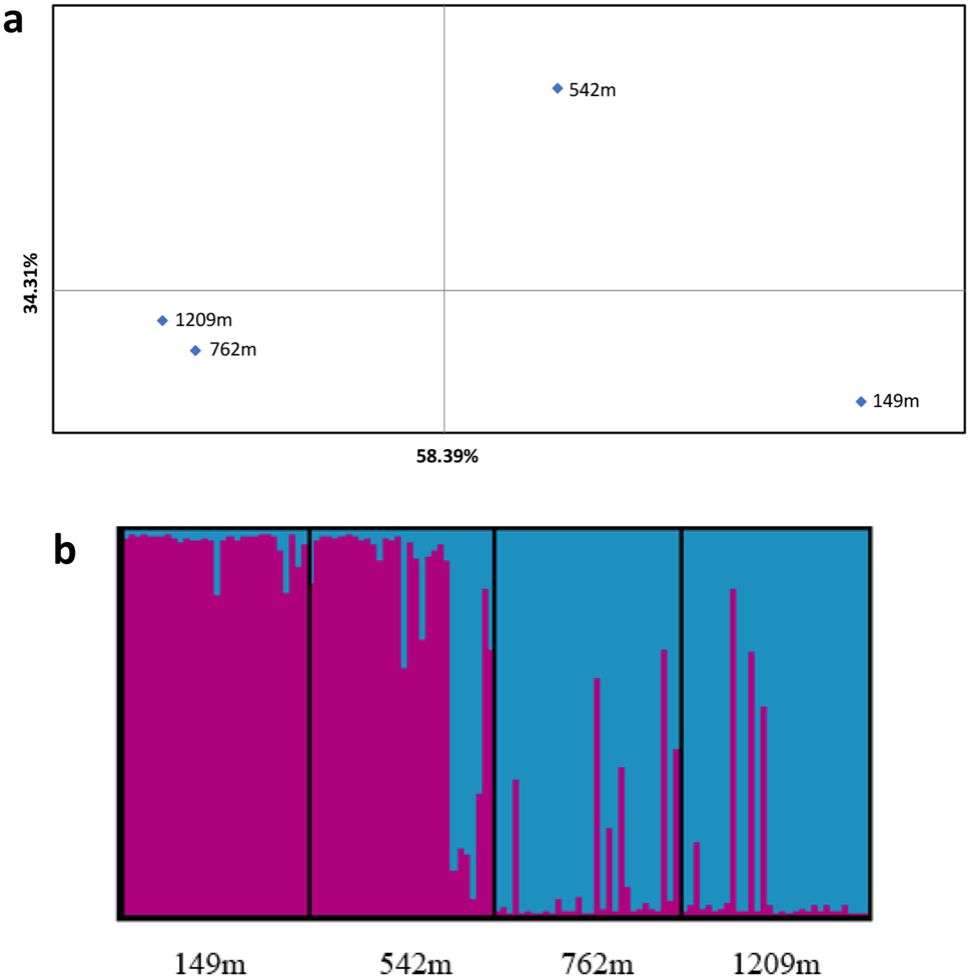
PCoA plot obtained from the *F*_*ST*_*P* matrix using GenAlEx (**a**) and graphical representation of the co-ancestry percentages obtained with Distruct 1.1. (**b**). In panel (**a**), highlighted populations are those showing a higher genetic correlation. In panel (**b**), each cluster is represented by a specific color and each bar represents a specific individual and its percentage of identity to the two clusters (K1 and K2).

The 11 SSR used to genotype the four populations of mosquitoes originating from different altitudes of Tirana and the Dajti mountain did not display signs of linkage disequilibrium between any pairs, suggesting that populations were independent, and their variability might well reflect genome-wide patterns. Across these loci, mosquito samples displayed similar levels of variability (Table S1) suggesting no recent bottleneck effect. However, a certain degree of inter-population differentiation was detected which increased along with altitude. Indeed, high *F*_*ST*_P values (0·107, 0·122 and 0·134) were estimated between the population of Tirana at 149 m and the others (542 m, 762 m and 1209 m, respectively; Table S2). The two populations at the highest altitudes (762 m and 1209 m) were more genetically similar to each other (*F*_*ST*_P value of 0·022) than with others. This differentiation pattern was confirmed from the PCoA (Fig. 1a) which separated the population 149 m from the two populations 762 m and 1209 m along the first axis (representative for 58·4% of variance). The population 542 m was at an intermediate position on the second axis (depictive for 34·3% of variance).

The Bayesian clustering analysis implemented in STRUCTURE, based on Evanno’s method, indicated that two lineages (K1 and K2, Fig. 1b) represented the most parsimonious partitioning of the ancestry among individuals from the four populations (Table S3). The population 149 m was mainly structured in K1 (0·972%); starting from this altitude, we observed a gradual transitioning in genetic composition towards the second lineage or K2. Indeed, the population 542 m displayed an ancestry profile structured for 0·799% in K1 and 0·201% in K2. At higher altitudes, the populations 762 m and 1209 m presented the highest membership in K2 (0·895% and 0·910% respectively, Fig. 1b). When comparing with other neighbouring populations in Europe, the four Albanian populations were genetically different from the other populations (Fig. S1). The population from Tirana was genetically related to mosquitoes from China while mosquitoes from Brescia in the region of Lombardy in Northern Italy were genetically close to mosquitoes from La Reunion. Surprisingly, the population from Cesena in the Emilia-Romagna region in Italy was a mix of mosquitoes from Tirana and La Reunion. Mosquitoes from Greece were genetically distinct from the other populations (Fig. S1).

### *Ae. albopictus* mosquitoes from high altitudes disseminate and transmit CHIKV

**Figure 2:**
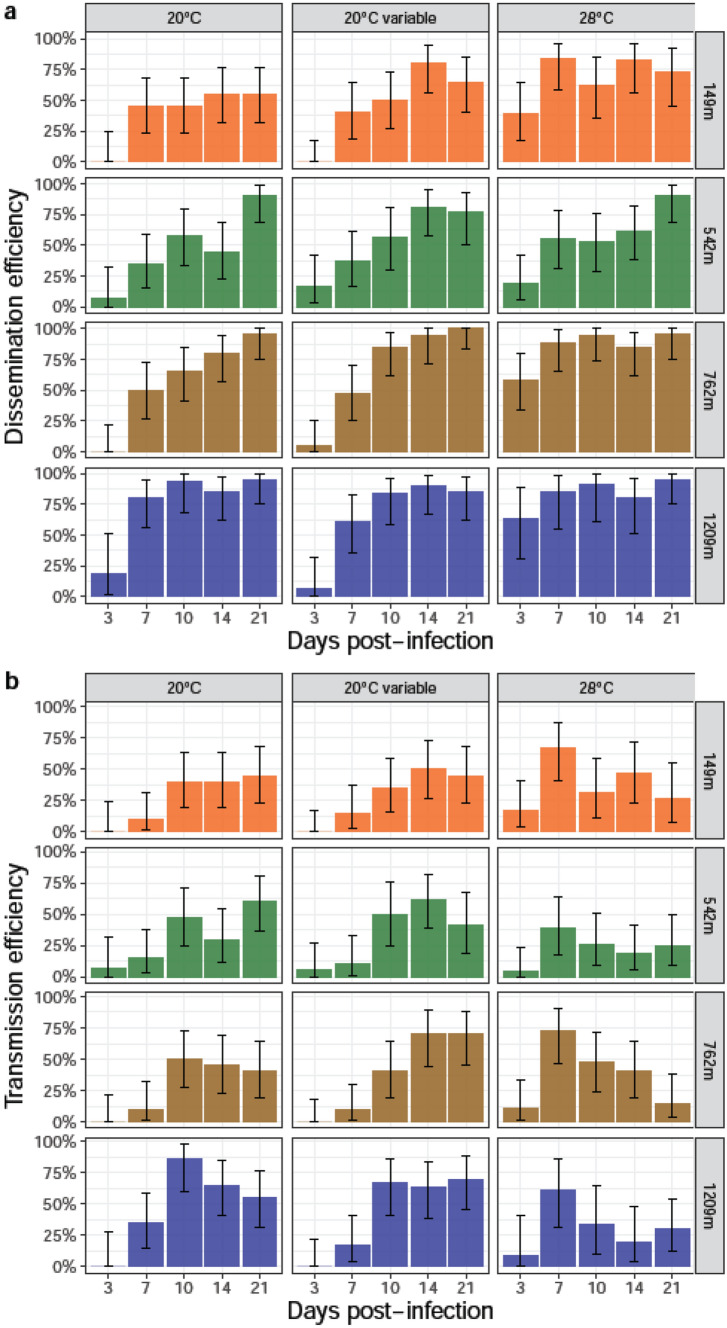
Dissemination and transmission efficiencies of *Ae. albopictus* populations infected with CHIKV examined at different days (3, 7, 10, 14, and 21) after incubation at different temperatures (20 °C, 20 °C variable, 28 °C). After infection, mosquito heads and saliva were titrated. (**a**) Dissemination efficiency corresponds to the proportion of mosquitoes with infected heads. (**b**) Transmission efficiency refers to the proportion of mosquitoes with infectious saliva. Error bars show the exact Binomial confidence interval (95%).

To examine whether viral dissemination and transmission varied according to the incubation temperature and the day post-infection (dpi), dissemination efficiency (DE) and transmission efficiency (TE) were estimated for each population infected with CHIKV (Fig. 2a,b; Table S4).

When considering DE, most populations started viral dissemination at 3 dpi though with overall low success rates except when mosquitoes were incubated at 28 °C (Fig. 2a). DEs were high whatever the population, reaching 83.33% for 149 m (7 dpi, 28 °C), 90% for 542 m (21 dpi, 20 °C or 28 °C), 100% for 762 m (21 dpi, 20 °C variable), and 95% for 1209 m (21 dpi, 20 °C or 28 °C) (Table S4). When comparing DEs obtained at the three incubation temperatures for each dpi and each population, only one comparison (population 762 m, 3 dpi; DE: 0% at 20 °C, 5.26% at 20 °C Var, and 57.89% at 28 °C) among 20 was statistically significant (after Bonferroni correction) (Fisher’s exact test: p < 0·05).

For TE, most populations started viral transmission from 3 dpi when incubated at 28 °C, and 7 dpi at 20 °C variable and 20 °C (except population 542 m at 3 dpi) (Fig. 2b). TEs were lower reaching 66.66% for 149 m (7 dpi, 28 °C), 61.90% for 542 m (14 dpi, 20 °C variable), 72.22% for 762 m (7 dpi, 28 °C), and 86.67% for 1209 m (10 dpi, 20 °C) (Table S4). When comparing TEs at the three incubation temperatures for each dpi and each population, two comparisons among 20 were significant (after Bonferroni correction) (Fisher’s exact test: p < 0·05): populations 149 m at 7 dpi and 762 m at 3 dpi. The efficiency of the salivary glands as barrier to viral transmission can be measured by estimating the transmission rate (TR). For each population, TRs were comparable whatever the dpi and the incubation temperature (Fisher’s exact test: p > 0·05) with TR reaching 80.0% for 149 m (7 dpi, 28 °C), 66.7% for 542 m (21 dpi, 20 °C), 81.2% for 762 m (7 dpi, 28 °C), and 92.9% for 1209 m (10 dpi, 20 °C) (Table S5). When examining the extrinsic incubation period (EIP), all four populations were able to transmit from 3 dpi when incubated at 28 °C and only one population (542 m) from 3 dpi at 20 °C and 20 °C Variable.

**Figure 3:**
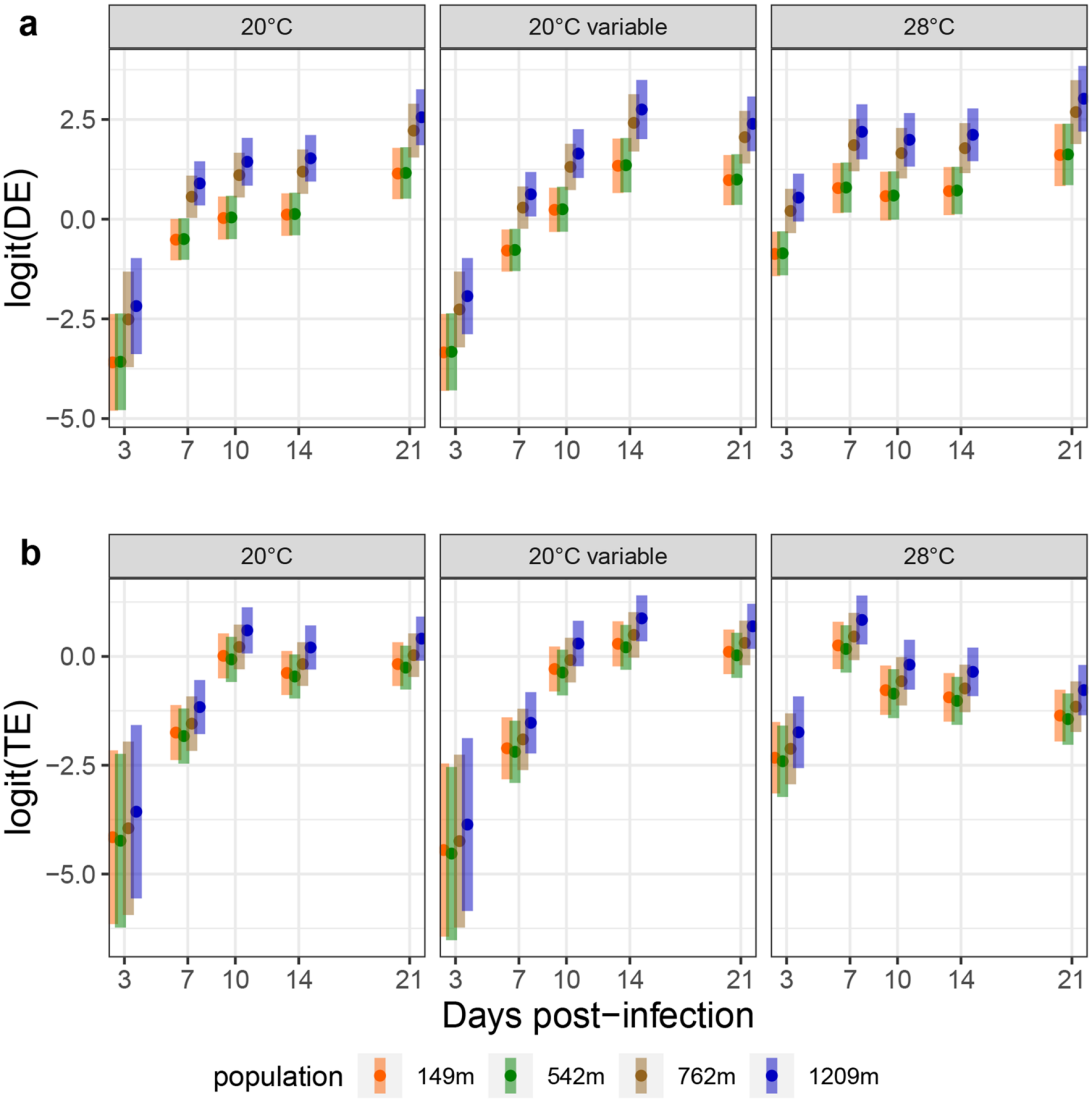
Predicted value of Dissemination Efficiency (DE, panel **a**) and Transmission Efficiency (TE, panel **b**) according to days-post infection, modeled using logistic regression. In both panels, graphs show the expected marginal means (dots) with corresponding 95% confidence intervals (bars) on the logit scale, highlighting the interacting effect of temperature on days post-infection.

In Fig. 2a, a sharp difference could be observed between the magnitudes of DE at 28 °C compared to that at lower temperatures, especially in the early dpi. This trend can be captured statistically by including interaction terms to the regression models, allowing the effect of incubation temperatures to vary according to mosquito populations. Figure 3 shows the predicted value of the outcome (Fig. 3a: DE; Fig. 3b: TE) on the logit scale according to dpi for the four populations. DE was favoured for populations at higher altitudes and an altitude cut-off was observed between populations 542 m and 762 m while no significant difference was identified between the two populations at highest altitudes and the two populations at lowest altitudes. The rate at which DE increased according to dpi was lower at 28 °C, reaching high values earlier after infection. For TE however, this cut-off was not found.

### No significantly different CHIKV loads in *Ae. albopictus* saliva

**Figure 4:**
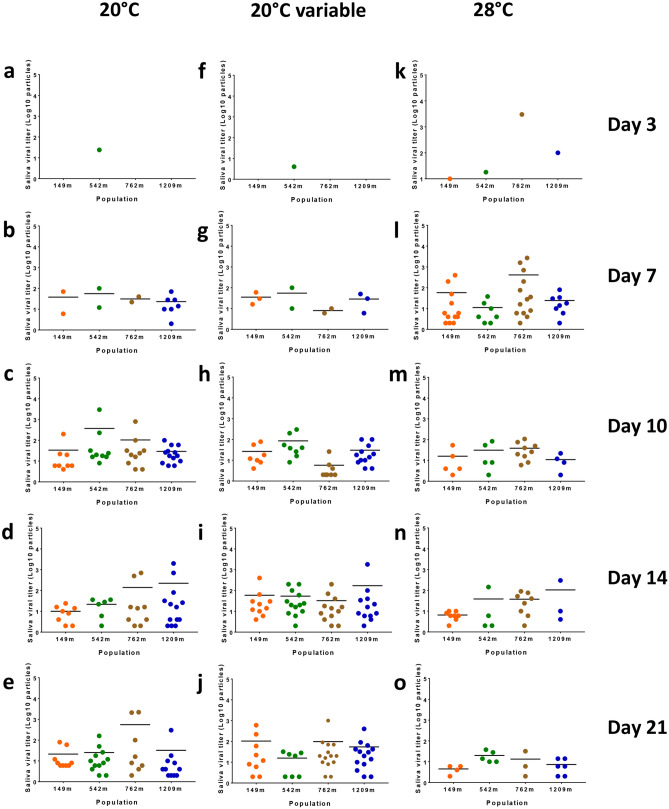
Viral titers in saliva of *Ae. albopictus* infected with CHIKV and examined at different days (3 (**a**,**f**,**k**), 7 (**b**,**g**,**l**), 10 (**c**,**h**,**m**), 14 (**d**,**l**,**n**), and 21(**e**,**j**,**o**)) after incubation at different temperatures (20 °C, 20 °C variable, 28 °C). Saliva was collected from individual females using the forced salivation technique and titrated on C6/36 *Ae. albopictus* cells. Bars indicate the mean.

To test whether the number of viral particles delivered by mosquitoes varied according to the incubation temperature and the dpi, saliva collected from each mosquito was titrated. When examining the four populations at a given dpi and incubation temperature, no significant difference of saliva viral titers was detected (Kruskal–Wallis test: p > 0·05; Fig. 4a-o). Likewise, no significant difference was detected according to the dpi when considering each population at a given incubation temperature (Table S6). The highest mean number of viral particles was detected in two mosquito saliva of the population 762 m incubated for 3 days at 28 °C: 10^3.18±3.32^ (1504 ± 2116) infectious particles.

### *Ae. albopictus* only disseminates and transmits DENV at 28 °C

**Figure 5:**
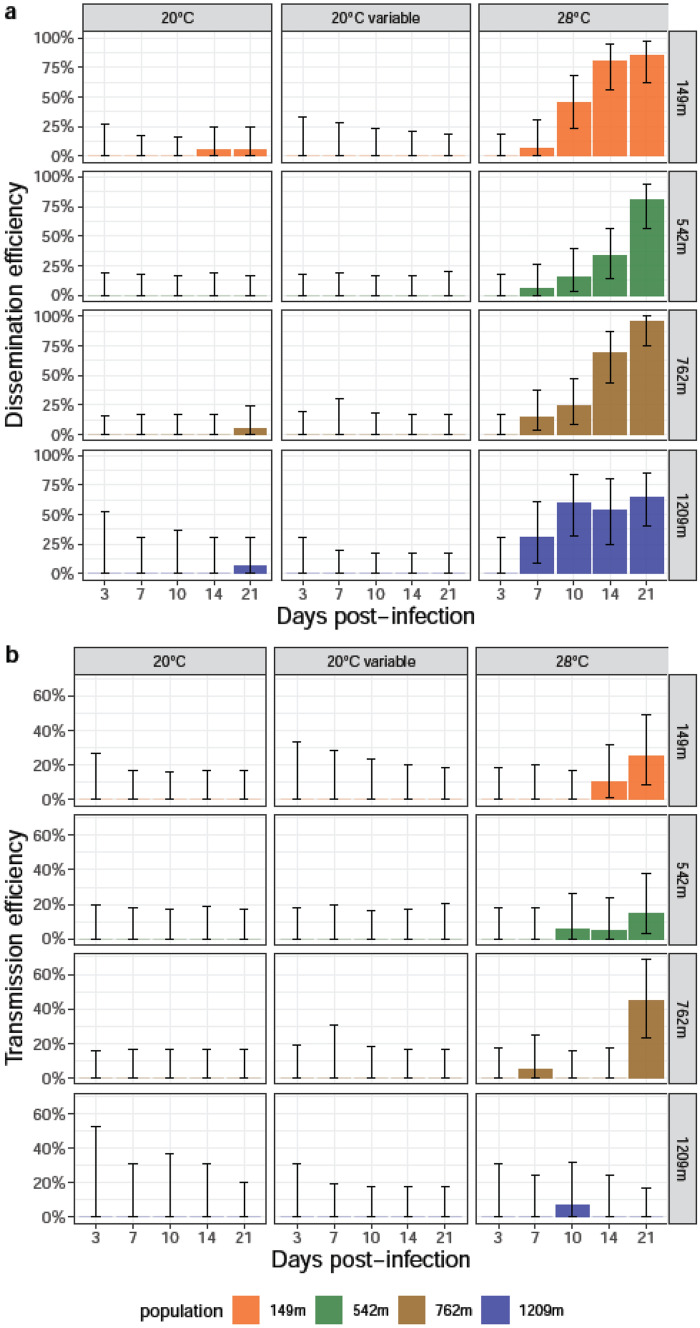
Dissemination and transmission efficiencies of *Ae. albopictus* populations infected with DENV examined at different days (3, 7, 10, 14, and 21) after incubation at different temperatures (20 °C, 20 °C variable, 28 °C). After infection, mosquito heads and saliva were titrated. (**a**) Dissemination efficiency corresponds to the proportion of mosquitoes with infected heads. (**b**) Transmission efficiency refers to the proportion of mosquitoes with infectious saliva. Error bars show the exact Binomial confidence interval (95%).

To measure the vector competence of *Ae. albopictus* to DENV, viral dissemination and transmission were estimated according to the incubation temperature and the dpi (Fig. 5a; Table S4). Viral dissemination was mainly detected at 28 °C, with DE starting from 7 dpi and increasing significantly along with dpi (Fisher’s exact test: p < 0·05) (Fig. 5a). DE reached the highest values at 21 dpi (85% for 149 m, 80% for 542 m, 94.73% for 762 m, and 65% for 1209 m). When comparing DEs obtained at the three incubation temperatures for each dpi and each population, 10 comparisons among 16 were statistically significant (after Bonferroni correction) (Fisher’s exact test: p < 0·05) with a viral dissemination mainly observed at 28 °C (Table S4). Consequently, viral transmission was only detected at 28 °C reaching highest TEs at 21 dpi (33.33% for 149 m, 15% for 542 m, 42.10% for 762 m) (Fig. 5b). No transmission was observed for population 1209 m at 21 dpi whatever the incubation temperature (Table S4). When comparing TE at the three incubation temperatures for each dpi and each population, only one among 8 comparisons was significantly different (after Bonferroni correction) (Fisher’s exact test: p < 0·05): population 762 m at 21 dpi (Table S4). When examining TR, values were low with a maximum of 42·10% for population 762 m (21 dpi, 28 °C), meaning that more than half of mosquitoes with disseminated infection were not able to transmit DENV (Table S5). When examining the EIP, all four populations only transmitted at 28 °C: 149 m from 14 dpi, 542 m from 10 dpi, 762 m from 7 dpi, and 1209 m at 10 dpi (Fig. S2). The highest mean number of viral particles detected in mosquito saliva was 10^2.04^ (110) infectious particles for one mosquito of the population 1209 m (10 dpi, 28 °C) (Table S6).

## Discussion

In this study, we showed that the four *Ae. albopictus* populations collected at 149 m, 542 m, 762 m, and 1209 m were able to transmit CHIKV at 20 °C as at 28 °C. Contrariwise, DENV transmission only occurs at 28 °C; we observed that transmission is less likely for the population at 1209 m altitude. These results indicate that CHIKV transmission may occur at high altitudes. So as the distribution of *Ae. albopictus* is expanding, the risk of CHIKV outbreaks can extend beyond South Europe and reach northern European countries.

Two waves of introduction of *Ae. albopictus* in Europe are mainly described: from China to Tirana in Albania in 1979^33^, and from Japan via the United States and La Reunion Island in Italy^34^. We showed that the two *Ae. albopictus* samples collected at high altitudes (762 m and 1209 m) in Dajti mountain in Central Albania were genetically different from the two populations at low altitudes (149 m and 542 m) and all four populations from Dajti mountain are genetically distinct from the population of Tirana^3^. It is believed that *Ae*. *albopictus* from Tirana experienced a different colonization dynamic than those from Italy, partly related to strong bottlenecks and/or differences of populations in the ability to adapt to local environmental conditions^35^; while the species spread rapidly and widely in Italy after its first detection in 1990, *Ae. albopictus* remains confined to Albania after its introduction in 1979.

The genetic variability of founder populations is expected to be extremely low as usually, only few individuals are introduced and among them, few survive^33^; this hypothesis could be raised to explain the significant genetic divergence between populations 149 m and 542 m with populations 762 m and 1209 m. The ability of *Ae. albopictus* to colonize high altitudes is presumably related to the cold hardiness of eggs. This phenotypic trait results in a modification of the egg endochorion^36^; a physical change in the chorion structure (more compact wax layers or additional inter-membrane space between different layers) protects the embryo against freezing. Epigenetic control of the expression of these genes may allow rapid adaptation to cold; small RNAs and histone modifications are involved in diapause mechanisms^36–38^. To note, we used the generation F6 for genotyping which could introduce some distortions in the genetic analysis.

Vector competence data is a reliable predictor for emergence of arboviral diseases as it has been demonstrated with *Ae. albopictus* populations from southern Europe^39^. As each pairing vector and virus genotypes lead to a specific outcome of infection which is also modulated by the temperature, all described as genotype-by-genotype-by-environment (G x G x E) interactions^40^, our study will feed a risk map of CHIKV outbreaks in Europe associated with *Ae. albopictus.* We show that all four *Ae. albopictus* populations transmit CHIKV at 20 °C and 28 °C and DENV only at 28 °C; these temperatures could serve as a proxy for the transmission of CHIKV and DENV at higher latitudes in Europe. To obtain adult mosquitoes of same age and size, immature stages and adults were exposed to a controlled temperature of 24 ± 1 °C before experimental infections. Lower temperature may generate small mosquitoes ingesting a lower amount of blood and then lower number of viral particles with subsequent effects on mosquito susceptibility to virus infection^41^. The different mosquito genetic backgrounds may have impacted competence for arboviruses^33^. Mosquitoes may regulate gene expression to adapt their phenotypes and maintain fitness in response to stressors such as temperature changes^42,43^. Temperature may alter mosquito gene expression including genes related to mosquito antiviral responses such as RNA interference^44^. RNA interference can be disrupted when mosquitoes are exposed to cooler temperatures^45^. Impairment of immune barriers may affect susceptibility to arboviruses including CHIKV^45,46^. Moreover, temperature may also affect mosquito microbial communities (protozoans, fungi, bacteria and viruses) which intervene in protection against pathogens^25^. Mosquito microbiota plays an important role in defining differences in vector competence^47^. At 20 °C, some commensal bacteria may promote arboviral infection by facilitating virus entry in the mosquito gut epithelium^48^.

As the main vector is *Ae. albopictus*, control strategies in Europe should be more feasible than in endemic regions where multiple genotypes and vectors are involved. Likewise, innovative mosquito control strategies can also be explored such as the use of trans-infected endosymbiotic *Wolbachia* bacteria^49^ and possibly lead to a successful control of *Ae. albopictus* as it has happened in the past for *Ae. aegypti*. Nevertheless, as temperature on the field may alter microbiota composition and mosquito immune responses during viral infections, the relevance and robustness of such method should be evaluating considering the different environmental temperatures^50^. Thus, the Albanian experience teaches us that chikungunya is no longer a tropical disease and its expansion will follow that of its vector. Active cooperation between European countries is critical in succeeding to control *Ae. albopictus* which does not recognize borders.

## Materials and methods

### Ethic statements

Animals (mice as blood source for mosquito rearing and rabbit for blood in mosquito experimental infections) were housed in the Institut Pasteur animal facilities (Paris) accredited by the French Ministry of Agriculture for performing experiments on live rodents. Work on animals was performed in compliance with French and European regulations on care and protection of laboratory animals (EC Directive 2010/63, French Law 2013-118, February 6th, 2013). All experiments were approved by the Ethics Committee #89 and registered under the reference APAFIS (Autorisation de Projet utilisant des Animaux à des FIns Scientifiques)#6573-201606l412077987 v2. The study was carried out in compliance with the ARRIVE guidelines.

### Mosquito rearing

**Figure 6:**
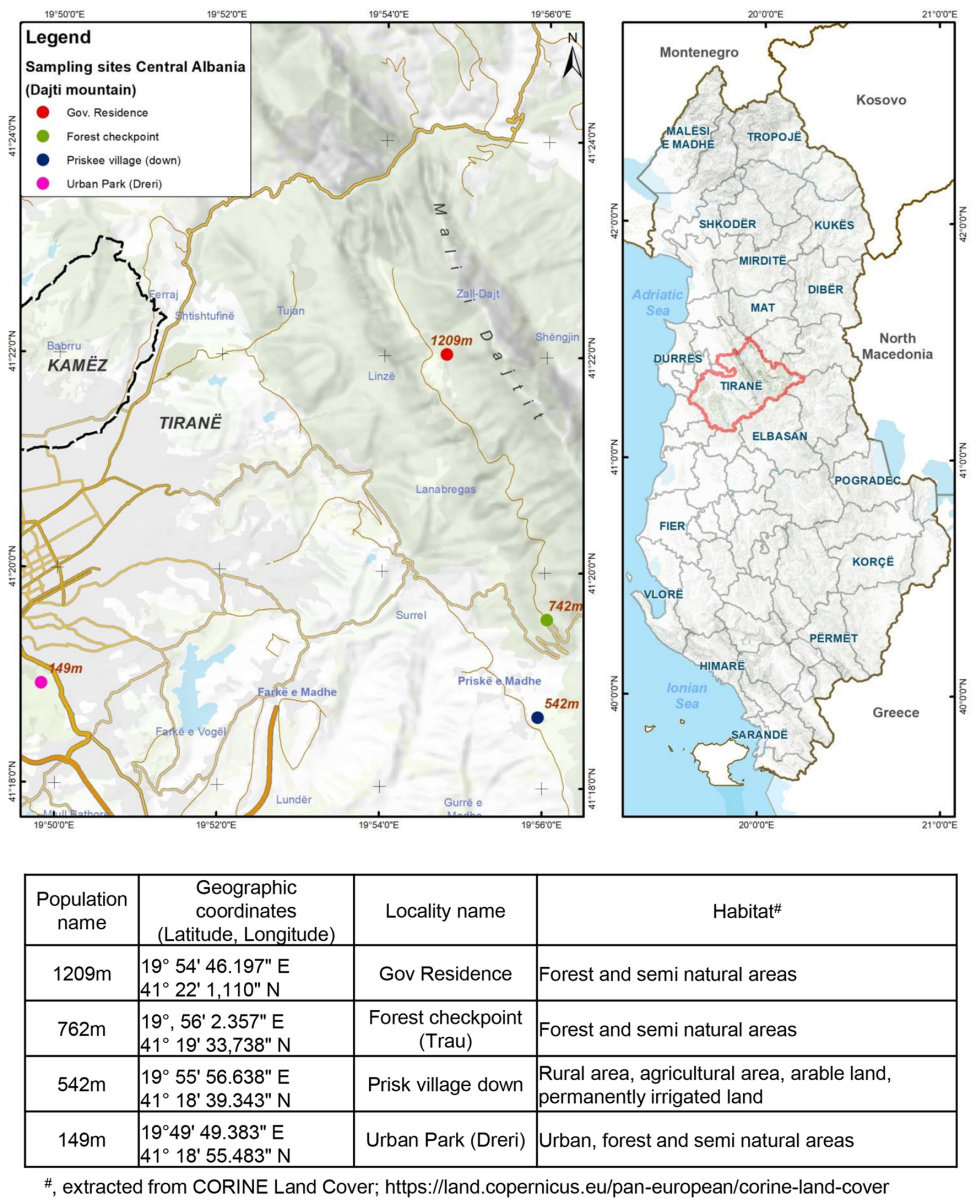
*Aedes albopictus* populations collected in Albania. Mosquitoes were processed for genotyping with microsatellites and vector competence studies to DENV and CHIKV. The figure was prepared by Eng. Migel Ali, GIS expert at the Company Geo Consulting Albania (https://gc-al.com/en/staff_mali.php).

*Aedes albopictus* eggs were collected in Central Albania at Dajti mountain located a few kilometers from Tirana, the capital city of Albania. Ovitraps were placed at four altitudes (149 m, 542 m, 762 m, and 1209 m) (Fig. 6) and eggs were weekly collected in 2015 during the period of optimal activities of *Ae. albopictus*^27^, from June to mid-August. Batches of eggs were sent to the Institut Pasteur and rearing was carried out in controlled conditions (24 ± 1 °C, 70% relative humidity, a 12:12 h (Light:Dark) photoperiod). Larvae were distributed in pans (200 larvae/pan) containing 1.5 L of dechlorinated tap water supplemented with yeast tablets. Obtained adults were placed in cages and daily provided with 10% sucrose solution. Generations F4/F5 mosquitoes were used for experimental infections and F6 for genotyping.

### Microsatellite genotyping of Ae. albopictus populations sampled at different altitudes

Genomic DNA was individually extracted from 120 mosquitoes (15 males and 15 females for each population) and genotyped at 11 polymorphic simple sequence repeats (SSR) loci: Aealbmic1, 2, 3, 5, 6, 9, 11, 14, 15, 16 and 17 as described in^51^. PCR amplifications and fragment identifications were performed as described in^52^. Each PCR product was then diluted 1:10 in ddH2O water and 2 μL of this dilution was added to 10 μL of a mixture of deionized formamide and GeneScan-500 ROX size standard (Applied Biosystems, CA, USA). Genotyping was processed in an ABI3730XL sequence analyser (Applied Biosystems) and data analysed using GeneScan and Genemapper software. The first step of the genetic analysis was to bin the genotyped raw data using TANDEM V. 1.09^53^; this program helps to overcome problems related with genotyping errors. When microsatellite amplification was not successful or scoring was uncertain, re-extraction of DNA was performed.

### Variability of mosquito populations and genetic structure analyses

The genetic variability of the four sampled populations was estimated in terms of mean number of alleles (*N*_*a*_), mean number of effective alleles (*N*_*e*_), observed and expected heterozygosity (*H*_*o*_ and *H*_*e*_), unbiased expected heterozygosity (*uH*_*e*_), inbreeding coefficient (F) and pairwise *F*_*ST*_ (*F*_*ST*_P) using GenAlEx 6.51^54^. The statistical significance of each *F*_ST_P value was assessed by comparison of the observed value with the values obtained in 10,000 matrix permutations. Linkage disequilibrium between pairs of loci in each sample and deviations from Hardy–Weinberg equilibrium (HWE) at each locus/sample combination were examined with GENEPOP V.4.7.5^55,56^ and the statistical significance was assessed following Bonferroni corrections. The relationships among populations were appraised using Principal Coordinate Analysis (PCoA) in GenAlEx^54^.

Genetic population structure was assessed using Bayesian clustering method proposed in the software STRUCTURE v.2.3.4^57^, using the admixture model and assuming independent allele frequencies. The burn-in was set to 500,000 steps and was followed by 1,000,000 Markov Chain Monte Carlo replications. All runs were repeated 20 times for each number of possible clusters (K), set between 1 to 8 (i.e. twice the number of populations). The proper number of genetic clusters was determined by plotting the log probability (L(K)) and ΔK across multiple runs as implemented in STRUCTURE HARVESTER (http://taylor0.biology.ucla.edu/structureHarvester/). The Greedy algorithm in CLUMPP V.1.1.2^58^ was used to merge the 20 independent runs and the graphical representation of the co-ancestry percentages obtained was plotted using DISTRUCT 1.1^59^. The four populations studied were compared to populations from neighbouring countries in Europe (Italy and Greece) and from China and La Reunion, both locations being the source of colonisation of Albania and Italy by *Ae. albopictus*, respectively^33^.

### Viral strains and mosquito experimental infections

Mosquito experimental infections used CHIKV 06.21 and DENV-2 strains. CHIKV 06.21 (accession number AM258992) isolated in 2005 from a patient on La Reunion belongs to the East-Central-South African (ECSA) lineage and contains the E1-A226V mutation^60^. DENV-2 strain (accession number: MK268692) was isolated in 1974 from a patient in Bangkok (Thailand)^61^. Both strains are our references for each virus. Viral stocks were produced after passages on C6/36 cells.

Batches of 6/10-day-old females were fed for 15 min through a pig intestine membrane covering the base of a feeder (Hemotek® membrane feeding system, UK) containing 1·4 mL of washed rabbit erythrocytes, 0·7 mL of viral suspension, and 10 mM of ATP. The titer of the blood-meal was 10^7^ FFU/mL for both viruses. Engorged females were transferred in cardboard containers and maintained with 10% sucrose in climatic chambers (KB 53, Binder, Tuttlingen, Germany) under three different incubation temperatures: (i) a constant temperature of 28 °C ± 0·1 °C, (ii) a constant temperature of 20 °C ± 0·1 °C or (iii) at temperatures displaying daily fluctuations between 17 °C ± 0·1 °C and 23 °C ± 0·1 °C (average: 20 °C ± 0·1 °C). The temperature of 28 °C represents a mean temperature in tropical regions and 20 °C, a mean temperature in France during the 2010 CHIKV outbreak^40^. These temperatures correspond to those recorded during the period of the year when mosquitoes laid the most eggs in ovitraps (Fig. S3). To limit confounding factors, mosquitoes (immature stages and adults) were reared at 24 ± 1 °C before experimental infections; adult females reared at 32 °C are larger and better transmit CHIKV than females at 18 °C^29^.

### Analysis of mosquito susceptibility

Batches of 20 females were analysed at 3, 7, 10, 14, 21 day post-infection (dpi). After cold anaesthesia, wings and legs of each mosquito were removed and the proboscis was inserted into 20 µL tip filled with 5 µL of Fetal Bovine Serum (FBS) for saliva collection^62^. After 30–45 min, medium containing saliva was expelled into 1.5 mL tube containing 45 µL of Leibovitz L15 medium (Invitrogen, CA, USA). Head and body were separated from each mosquito and ground individually in 300 µL of L15 medium supplemented with 3% FBS.

For saliva and head samples, infectious particles were detected using focus fluorescent assay on C6/36 *Ae. albopictus* cells. Samples were inoculated onto monolayers of C6/36 *Ae. albopictus* cell culture in 96-well plates. After incubation at 28 °C for 3 and 5 days for CHIKV and DENV respectively, plates were fixed with 10% formaldehyde, washed and stained using hyper-immune ascetic fluid as primary antibody and Alexa Fluor 488 goat anti-mouse IgG as the second antibody (Life technologies, CA, USA).

Two parameters were used to describe the viral dissemination and transmission. Dissemination efficiency (DE;^63^) gives the proportion of mosquitoes with infected head (i.e. mosquitoes able to disseminate the virus from the midgut into the mosquito general cavity). Transmission efficiency (TE;^63^) refers to the proportion of mosquitoes with infectious saliva (i.e. mosquitoes with virus having succeeded in replicating in salivary glands and released with saliva during mosquito blood feeding). Viral loads in mosquito saliva were estimated. To measure whether the salivary glands can act as a barrier to the release of viral particles in saliva, the transmission rate (TR) was also calculated, which corresponds to the proportion of mosquitoes with virus detected in saliva among mosquitoes with disseminated virus in head. Moreover, the extrinsic incubation period (EIP) was calculated, which refers to the time from ingestion of an infectious blood meal to transmission of virus.

### Statistical analysis

Logistic regressions were used to model DE and TE according to mosquito populations, incubation temperatures, and dpi. All three covariates (mosquito population, temperature, and dpi) were coded as categorical and for each, the lowest value served as reference level (*i.e.* 20 °C constant, 149 m and 3 dpi). Akaike Information Criterion was used as a guide for model selection, investigating combinations of fixed effects and interactions between the three covariates of interest. The low dimensionality of the data also allowed for conveniently visualizing the possible interactions of covariates over two-dimensions graphics (outcome according to dpi) stratified over incubation temperatures and mosquito populations.

Statistical tests were conducted using the STATA software (StataCorp LP, Texas, USA) and R 4.0.3. P-values above 0·05 were considered non-significant. If necessary, the significance level of each test was adjusted based on the number of tests run, according to the sequential method of Bonferroni^64^ or using Tukey’s range test for multiple comparisons in generalized linear models (R package emmeans 1.5.2–1;^65^).

## Supplementary Information

Supplementary Information.

## References

[1] Barzon L (2018). Ongoing and emerging arbovirus threats in Europe. J. Clin. Virol..

[2] Papa A (2019). Emerging arboviruses of medical importance in the Mediterranean region. J. Clin. Virol..

[3] Adhami J, Reiter P (1998). Introduction and establishment of Aedes (Stegomyia) albopictus skuse (Diptera: Culicidae) in Albania. J. Am. Mosq. Control Assoc..

[4] Bonizzoni M, Gasperi G, Chen X, James AA (2013). The invasive mosquito species Aedes albopictus: Current knowledge and future perspectives. Trends Parasitol..

[5] Rezza G (2007). Infection with chikungunya virus in Italy: An outbreak in a temperate region. Lancet.

[6] La Ruche G (2010). First two autochthonous dengue virus infections in metropolitan France, September 2010. Euro Surveill..

[7] Giron S (2019). Vector-borne transmission of Zika virus in Europe, southern France, August 2019. Euro Surveill..

[8] Angelini R (2007). An outbreak of chikungunya fever in the province of Ravenna, Italy. Euro Surveill..

[9] Tsetsarkin KA, Vanlandingham DL, McGee CE, Higgs S (2007). A single mutation in chikungunya virus affects vector specificity and epidemic potential. PLoS Pathog..

[10] Vazeille M (2007). Two Chikungunya isolates from the outbreak of La Reunion (Indian Ocean) exhibit different patterns of infection in the mosquito Aedes albopictus. PLoS ONE.

[11] Anon (1928). The dengue epidemic in Greece. League Nations Monthly Epidemiol. Rep..

[12] Curtin TJ (1967). Status of Aedes aegypti in the Eastern Mediterranean. J. Med. Entomol..

[13] Kotsakiozi P, Gloria-Soria A, Schaffner F, Robert V, Powell JR (2018). Aedes aegypti in the Black Sea: Recent introduction or ancient remnant?. Parasit. Vectors.

[14] Gjenero-Margan I (2011). Autochthonous dengue fever in Croatia, August-September 2010. Euro Surveill..

[15] Marchand E (2013). Autochthonous case of dengue in France, October 2013. Euro Surveill..

[16] Giron S (2015). Nouvelles apparitions de cas autochtones de dengue en région Provence-Alpes-Côte d’Azur, France, août-septembre 2014. Bull. Epidémiol. Hebd..

[17] Succo T (2016). Autochthonous dengue outbreak in Nimes, South of France, July to September 2015. Euro Surveill..

[18] Terrien E (2019). Surveillance du chikungunya, de la dengue et du virus Zika en France métropolitaine, 2018. Bull. Epidémiol. Hebd..

[19] Santé Publique France. Chikungunya, dengue et zika - Données de la surveillance renforcée en France métropolitaine en 2020. (2020). <https://www.santepubliquefrance.fr/maladies-et-traumatismes/maladies-a-transmission-vectorielle/chikungunya/articles/donnees-en-france-metropolitaine/chikungunya-dengue-et-zika-donnees-de-la-surveillance-renforcee-en-france-metropolitaine-en-2019>.

[20] Delisle E (2015). Chikungunya outbreak in Montpellier, France, September to October 2014. Euro Surveill..

[21] Calba C (2017). Preliminary report of an autochthonous chikungunya outbreak in France, July to September 2017. Euro Surveill..

[22] Venturi G (2017). Detection of a chikungunya outbreak in Central Italy, August to September 2017. Euro Surveill..

[23] Jourdain F (2020). From importation to autochthonous transmission: Drivers of chikungunya and dengue emergence in a temperate area. PLoS Negl. Trop. Dis..

[24] Tisseuil C (2018). Forecasting the spatial and seasonal dynamic of Aedes albopictus oviposition activity in Albania and Balkan countries. PLoS Negl. Trop. Dis..

[25] Bellone R, Failloux AB (2020). The role of temperature in shaping mosquito-borne viruses transmission. Front. Microbiol..

[26] Mohammed A, Chadee DD (2011). Effects of different temperature regimens on the development of Aedes aegypti (L.) (Diptera: Culicidae) mosquitoes. Acta Trop..

[27] Prudhomme J (2019). The native European Aedes geniculatus mosquito species can transmit chikungunya virus. Emerg. Microbes Infect..

[28] Briegel H (1990). Fecundity, metabolism, and body size in Anopheles (Diptera: Culicidae), vectors of malaria. J. Med. Entomol..

[29] Westbrook CJ, Reiskind MH, Pesko KN, Greene KE, Lounibos LP (2010). Larval environmental temperature and the susceptibility of Aedes albopictus Skuse (Diptera: Culicidae) to Chikungunya virus. Vector Borne Zoonotic Dis..

[30] Alto BW, Reiskind MH, Lounibos LP (2008). Size alters susceptibility of vectors to dengue virus infection and dissemination. Am. J. Trop. Med. Hyg..

[31] Price DP, Schilkey FD, Ulanov A, Hansen IA (2015). Small mosquitoes, large implications: crowding and starvation affects gene expression and nutrient accumulation in Aedes aegypti. Parasit. Vectors.

[32] Liu-Helmersson J (2016). Climate change and aedes vectors: 21st century projections for dengue transmission in Europe. EBioMedicine.

[33] Vega-Rua A (2020). Vector competence of Aedes albopictus populations for chikungunya virus is shaped by their demographic history. Commun. Biol..

[34] Manni M (2017). Genetic evidence for a worldwide chaotic dispersion pattern of the arbovirus vector *Aedes albopictus*. PLoS Negl Trop Dis.

[35] Pichler V (2019). Complex interplay of evolutionary forces shaping population genomic structure of invasive Aedes albopictus in southern Europe. PLoS Negl. Trop. Dis..

[36] Kress A, Kuch U, Oehlmann J, Muller R (2016). Effects of diapause and cold acclimation on egg ultrastructure: new insights into the cold hardiness mechanisms of the Asian tiger mosquito Aedes (Stegomyia) albopictus. J. Vector Ecol..

[37] Reynolds JA, Bautista-Jimenez R, Denlinger DL (2016). Changes in histone acetylation as potential mediators of pupal diapause in the flesh fly Sarcophaga bullata. Insect Biochem. Mol. Biol..

[38] Poupardin R (2015). Early transcriptional events linked to induction of diapause revealed by RNAseq in larvae of drosophilid fly Chymomyza costata. BMC Genom..

[39] Mariconti M (2019). Estimating the risk of arbovirus transmission in Southern Europe using vector competence data. Sci. Rep..

[40] Zouache K (2014). Three-way interactions between mosquito population, viral strain and temperature underlying chikungunya virus transmission potential. Proc. Biol. Sci. R. Soc..

[41] Alto BW, Bettinardi D (2013). Temperature and dengue virus infection in mosquitoes: Independent effects on the immature and adult stages. Am. J. Trop. Med. Hyg..

[42] Pigllucci M (1996). How organisms respond to environmental changes: From phenotypes to molecules (and vice versa). Trends Ecol. Evol..

[43] Sorensen JG (2020). Pronounced plastic and evolutionary responses to unpredictable thermal fluctuations in drosophila simulans. Front. Genet..

[44] Blair CD, Olson KE (2015). The role of RNA interference (RNAi) in arbovirus-vector interactions. Viruses.

[45] Adelman ZN (2013). Cooler temperatures destabilize RNA interference and increase susceptibility of disease vector mosquitoes to viral infection. PLoS Negl. Trop. Dis..

[46] Kay BH, Jennings CD (2002). Enhancement or modulation of the vector competence of Ochlerotatus vigilax (Diptera: Culicidae) for ross river virus by temperature. J. Med. Entomol..

[47] Cirimotich CM, Ramirez JL, Dimopoulos G (2011). Native microbiota shape insect vector competence for human pathogens. Cell Host Microbe.

[48] Wu P (2019). A gut commensal bacterium promotes mosquito permissiveness to arboviruses. Cell Host Microbe.

[49] Moretti R (2018). Combining Wolbachia-induced sterility and virus protection to fight Aedes albopictus-borne viruses. PLoS Negl. Trop. Dis..

[50] Zouache K, Michelland RJ, Failloux AB, Grundmann GL, Mavingui P (2012). Chikungunya virus impacts the diversity of symbiotic bacteria in mosquito vector. Mol. Ecol..

[51] Houe V, Bonizzoni M, Failloux AB (2019). Endogenous non-retroviral elements in genomes of Aedes mosquitoes and vector competence. Emerg. Microbes Infect..

[52] Manni M (2015). Molecular markers for analyses of intraspecific genetic diversity in the Asian Tiger mosquito Aedes albopictus. Parasit. Vectors.

[53] Matschiner M, Salzburger W (2009). TANDEM: Integrating automated allele binning into genetics and genomics workflows. Bioinformatics.

[54] Peakall R, Smouse PE (2012). GenAlEx 6.5: Genetic analysis in Excel. Population genetic software for teaching and research—an update. Bioinformatics.

[55] Raymond M, Rousset F (1995). GENEPOP (Version 1.2): Population genetics software for exact tests and ecumenicism. J. Hered..

[56] Rousset F (2008). genepop'007: A complete re-implementation of the genepop software for Windows and Linux. Mol. Ecol. Resour..

[57] Pritchard JK, Stephens M, Donnelly P (2000). Inference of population structure using multilocus genotype data. Genetics.

[58] Jakobsson M, Rosenberg NA (2007). CLUMPP: A cluster matching and permutation program for dealing with label switching and multimodality in analysis of population structure. Bioinformatics.

[59] Rosenberg N (2004). DISTRUCT: A program for the graphical display of population structure. Mol. Ecol. Notes.

[60] Schuffenecker I (2006). Genome microevolution of chikungunya viruses causing the Indian Ocean outbreak. PLoS Med..

[61] Vazeille-Falcoz M, Mousson L, Rodhain F, Chungue E, Failloux A-B (1999). Variation in oral susceptibility to dengue type 2 virus of populations of Aedes aegypti from the islands of Tahiti and Moorea, French Polynesia. Am. J. Trop. Med. Hyg..

[62] Dubrulle M, Mousson L, Moutailler S, Vazeille M, Failloux A-B (2009). Chikungunya virus and Aedes mosquitoes: Saliva is infectious as soon as two days after oral infection. PLoS ONE.

[63] Vega-Rua A, Zouache K, Girod R, Failloux AB, Lourenco-de-Oliveira R (2014). High level of vector competence of *Aedes aegypti* and *Aedes albopictus* from ten American countries as a crucial factor in the spread of Chikungunya virus. J Virol.

[64] Holm S (1979). A simple sequentially rejective multiple test procedure. Scand. J. Stat..

[65] emmeans: Estimated Marginal Means, aka Least-Squares Means. R package version 1.5.2–1. (2020).

