# Supplementary material for: Impact of temperature on dengue and chikungunya transmission by the mosquito Aedes albopictus
Source: Sci Rep. 2022 Apr 28;12:6973. doi: 10.1038/s41598-022-10977-4 (PMC9051100; doi:10.1038/s41598-022-10977-4)
Supplement: Supplementary file 1 — Supplementary Information. [file 41598_2022_10977_MOESM1_ESM.doc]

**Impact of temperature on dengue and chikungunya transmission by the mosquito *Aedes albopictus***

Aurélien Mercier^1,£^, Thomas Obadia^2,3^, Davide Carraretto^4^, Enkelejda Velo^5^, Gaelle Gabiane^6^, Silvia Bino^5^, Marie Vazeille^6^, Giuliano Gasperi^4^, Catherine Dauga^6^, Anna R. Malacrida^4^, Paul Reiter^1,#^, Anna-Bella Failloux^6,#,*^

^1^Insects and Infectious Diseases Unit, Institut Pasteur, F-75015 Paris, France

^2^Bioinformatics and Biostatistics Hub, Institut Pasteur, F-75015 Paris, France

^3^Institut Pasteur, Université de Paris, G5 Infectious Disease Epidemiology and Analytics, Department of Global Health, F-75015 Paris, France

^4^University of Pavia, Department of Biology and Biotechnology, Pavia, Italy

^5^Institute of Public Health, Tirana, Albania

^6^Institut Pasteur, Université de Paris, Unit of Arboviruses and Insect Vectors, Department of Virology, F-75015 Paris, France

^£^Current address: INSERM, Univ. Limoges, CHU Limoges, IRD, U1094 Neuroépidémiologie Tropicale, Institut d’Epidémiologie et de Neurologie Tropicale, GEIST, Limoges, France

^#^Co-last authors

^*^Correspondence: [anna-bella.failloux@pasteur.fr](mailto:anna-bella.failloux@pasteur.fr)

**Fig. S1. Genetic differentiation of *Ae. albopictus* populations based on the analysis of 11 microsatellite loci.** Ten populations were analyzed: four populations from Albania (our study) in comparison with six from other European countries (Xiamen from China, St. Pierre from La Reunion Island, Tirana from Albania, Brescia and Cesena from Italy, and Athens from Greece; data extracted from (49)).

**Figure S2. Viral titers in saliva of *Ae. albopictus* infected with DENV and examined at different days (3 (a, f, k), 7 (b, g, l), 10 (c, h, m), 14 (d, l ,n), and 21(e, j, o)) after incubation at different temperatures (20°C, 20°C variable, 28°C).** Saliva were collected from individual females using the forced salivation technique and titrated on C6/36 *Ae. albopictus* cells. Bars indicate the mean.

**Fig. S3. Mean temperatures recorded and mean number of eggs collected in the four sampling sites in 2013-2014:** (a) population 149m, (b) population 542m, (c) population 762m, and (d) population 1227m.

**
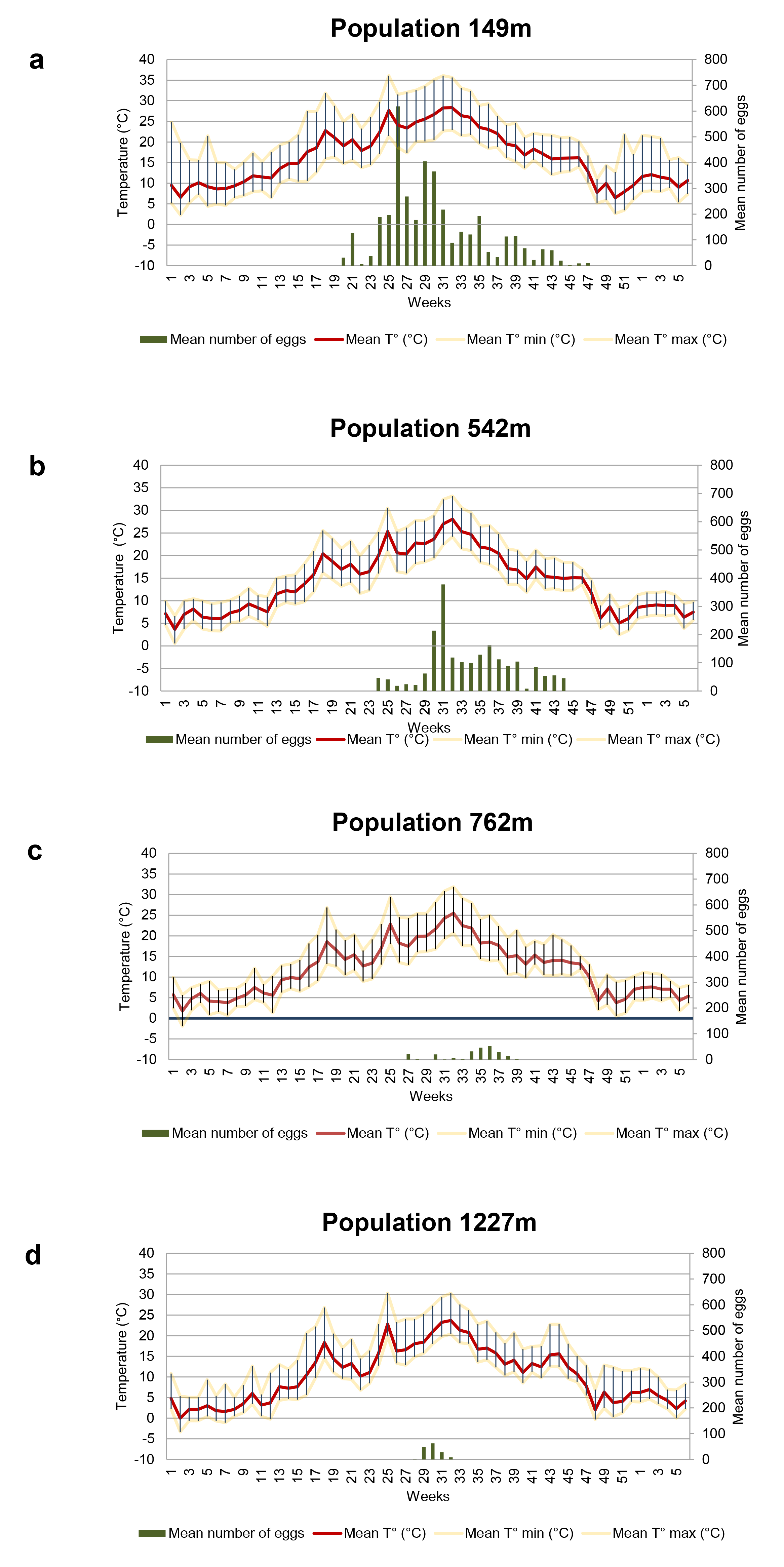
**

**Table S1. Estimation of genetic variability among the four populations of *Ae. albopictus* from Dajti Mountain in Albania.**

| Population | ***Na*** | ***Ne*** | ***Ho*** | ***He*** | ***uHe*** | **F** |
| --- | --- | --- | --- | --- | --- | --- |
| 149m | 3.545 | 2.049 | 0.316 | 0.394 | 0.401 | 0.272 |
| 542m | 4.091 | 2.243 | 0.312 | 0.414 | 0.421 | 0.176 |
| 762m | 3.364 | 2.028 | 0.312 | 0.403 | 0.410 | 0.223 |
| 1209m | 3.455 | 1.934 | 0.297 | 0.384 | 0.391 | 0.224 |

*N_a_*, mean number of alleles; *N_e_*, mean number of effective alleles; *H_o_*, observed heterozygosity; *H_e_*, expected heterozygosity; *uH_e_*, unbiased expected heterozygosity; F, inbreeding coefficient.

**Table S2. Pairwise *F_st_* matrix of the four *Ae. albopictus* populations collected in Dajti Mountain**. *F_ST_* values highlighted in dark red are the most significantly different from 0 (P < 10^-4^) after Bonferroni correction.

| 149m | 542m | 762m | 1209m | Population |
| --- | --- | --- | --- | --- |
| 0.000 |  |  |  | 149m |
| 0.107 | 0.000 |  |  | 542m |
| 0.122 | 0.096 | 0.000 |  | 762m |
| 0.134 | 0.092 | 0.022 | 0.000 | 1209m |

**Table S3**. **Average coefficient of ancestry obtained from a STRUCTURE run with K = 2 for 120 individuals of *Ae. albopictus* from four populations collected at different altitudes.**

| Dajti mountain | **K1** | **K2** |
| --- | --- | --- |
| 149m | 0.972 | 0.028 |
| 542m | 0.799 | 0.201 |
| 762m | 0.105 | 0.895 |
| 1209m | 0.090 | 0.910 |

**Table S4. Dissemination and Transmission efficiencies (%) of four *Ae. albopictus* populations from Albania (149m, 542m, 762m, 1209m) infected with CHIKV and** DENV, then incubated at three different temperatures (20°C, 20°C variable, and 28°C) and examined at five days post-infection (3, 7, 10, 14, and 21).

|  | | | **CHIKV** | | | | |  | **DENV** | | | | |
| --- | --- | --- | --- | --- | --- | --- | --- | --- | --- | --- | --- | --- | --- |
| **149m** |  | | 3 | 7 | 10 | 14 | 21 |  | 3 | 7 | 10 | 14 | 21 |
|  | DE | 20°C | 0%  (13; 0-24.7) | 45%  (20; 23.1-68.5) | 45%  (20; 23.1-68.5) | 55%  (20; 31.5-76.9) | 55%  (20; 31.5-76.9) |  | 0%  (12; 0-26.5) | 0%  (20; 0-16.8) | 0%  (21; 0-16.1) | 5%  (20; 0.1-24.9) | 5%  (20; 0.1-24.9) |
|  |  | 20°C Var | 0%  (20; 0-16.8) | 40%  (20; 19.1-63.9) | 50%  (20; 27.2-72.8) | 80%  (20; 56.3-94.3) | 65%  (20; 40.8-84.6) |  | 0%  (9; 0-33.6) | 0%  (11; 0-28.5) | 0%  (14; 0-23.2) | 0%  (16; 0-20.6) | 0%  (18; 0-18.5) |
|  |  | 28°C | 38.88%  (18; 17.3-64.3) | 83.33%  (18; 58.6-96.4) | 62.5%  (16; 35.4-84.8) | 82.35%  (17; 56.6-96.2) | 73.33%  (15; 44.9-92.2) |  | 0%  (18; 0-18.5) | 6.25%  (16; 0.2-30.2) | 45%  (20; 23.1-68.5) | 80%  (20; 56.3-94.3) | 85%  (20; 62.1-96.8) |
|  |  |  |  |  |  |  |  |  |  |  |  |  |  |
|  | TE | 20°C | 0%  (13; 0-24.7) | 10%  (20; 1.2-31.7) | 40%  (20; 19.1-63.9) | 40%  (20; 19.1-63.9) | 45%  (20; 23.1-68.5) |  | 0%  (12; 0-26.5) | 0%  (20; 0-16.8) | 0%  (21; 0-16.1) | 0%  (20; 0-16.8) | 0%  (20; 0-16.8) |
|  |  | 20°C Var | 0%  (20; 0-16.8) | 15%  (20; 3.2-37.9) | 35%  (20; 15.4-59.2) | 50%  (20; 27.2-72.8) | 45%  (20; 23.1-68.5) |  | 0%  (9; 0-33.6) | 0%  (11; 0-28.5) | 0%  (14; 0-23.2) | 0%  (16; 0-20.6) | 0%  (18; 0-18.5) |
|  |  | 28°C | 16.66%  (18; 3.6-41.4) | 66.66%  (18; 41.0-86.7) | 31.25%  (16; 11.0-58.7) | 47.05%  (17; 23.0-72.2) | 26.66%  (15; 7.8-55. 1) |  | 0%  (18; 0-18.5) | 0%  (16; 0-20.6) | 0%  (20; 0-16.8) | 10%  (20; 1.2-31.7) | 33.33%  (15; 8.7-49.1) |
|  | | | | | | | | | | | | | |
| **542m** | DE | 20°C | 6.66%  (15; 0.2-31.9) | 35%  (20; 15.4-59.2) | 57.87%  (19; 33.5-79.7) | 45%  (20; 23.1-68.5) | 90%  (20; 68.3-98.8) |  | 0%  (17; 0-19.5) | 0%  (1; 0-17.6) | 0%  (20; 0-16.8) | 0%  (18; 0-18.5) | 0%  (20; 0-16.8) |
|  |  | 20°C Var | 16.66%  (18; 3.6-41.4) | 36.84%  (19; 16.3-61.6) | 56.25%  (16; 29.9-80.2) | 80.95%  (21; 58.1-94.6) | 76.47%  (17; 50.1-93.2) |  | 0%  (19; 0-17.6) | 0%  (17; 0-19.5) | 0%  (21; 0-16.1) | 0%  (20; 0-16.8) | 0%  (16; 0-20.6) |
|  |  | 28°C | 19.04%  (21; 5.4-41.9) | 55.55%  (18; 30.8-78.5) | 52.63%  (19; 28.90-75.6) | 61.90%  (21; 38.4-81.9) | 90%  (20; 68.3-98.8) |  | 0%  (19; 0-17.6) | 5.26%  (19; 0.1-26.0) | 15.78%  (19; 3.4-39.6) | 33.33%  (21; 14.6-57.0) | 80%  (20; 56.3-94.3) |
|  |  |  |  |  |  |  |  |  |  |  |  |  |  |
|  | TE | 20°C | 6.66%  (15; 0.2-31.9) | 15%  (20; 3.2-37.9) | 47.36%  (19; 24.4-71.1) | 30%  (20; 11.9-54.3) | 60%  (20; 36.1-80.9) |  | 0%  (17; 0-19.5) | 0%  (19; 0-17.6) | 0%  (20; 0-16.8) | 0%  (18; 0-18.5) | 0%  (20; 0-16.8) |
|  |  | 20°C Var | 5.55%  (18; 0.1-27.3) | 10.52%  (19; 1.3-33.1) | 50%  (16; 24.7-75.3) | 61.90%  (21; 38.4-81.9) | 41.17%  (17; 18.4-67.1) |  | 0%  (19; 0-17.6) | 0%  (17; 0-19.5) | 0%  (2; 0-16.1) | 0%  (20; 0-16.8) | 0%  (16; 0-20.6) |
|  |  | 28°C | 4.76%  (21; 0.1-23.8) | 38.88%  (18; 17.3-64.3) | 26.31%  (19; 9.1-51.2) | 19.04%  (21; 5.4-41.9) | 25%  (20; 8.7-49.1) |  | 0%  (19; 0-17.6) | 0%  (19; 0-17.6) | 5.26%  (19; 0.1-26.0) | 4.76%  (21; 0.1-23.8) | 15%  (20; 3.2-37.9) |
|  | | | | | | | | | | | | | |
| **762m** | DE | 20°C | 0%  (15; 0-21.8) | 50%  (20; 27.2-72.8) | 65%  (20; 40.8-84.6) | 80%  (20; 56.3-94.3) | 95%  (20; 75.1-99.9) |  | 0%  (21; 0-16.1) | 0%  (20; 0-16.8) | 0%  (20; 0-16.8) | 0%  (20; 0-16.8) | 5%  (20; 0.1-24.9) |
|  |  | 20°C Var | 5.26%  (19; 0.1-26.0) | 47.61%  (21; 25.7-70.2) | 85%  (20; 62.1-96.8) | 94.11%  (17; 71.3-99.9) | 100%  (20; 83.2-100.0) |  | 0%  (17; 0-19.5) | 0%  (10; 0-30.8) | 0%  (18; 0-18.5) | 0%  (20; 0-16.8) | 0%  (20; 0-16.8) |
|  |  | 28°C | 57.89%  (19; 33.5-79.7) | 88.88%  (18; 65.3-98.6) | 94.73%  (19; 74.0-99.9) | 85%  (20; 62.1-96.8) | 95%  (20; 75.1-99.9) |  | 0%  (19; 0-17.6) | 15%  (20; 3.2-37.9) | 23.80%  (2; 8.2-47.2) | 68.42%  (19; 43.4-87.4) | 94.73%  (19; 75.1-99.9) |
|  |  |  |  |  |  |  |  |  |  |  |  |  |  |
|  | TE | 20°C | 0%  (15; 0-21.8) | 10%  (20; 1.2-31.7) | 50%  (20; 27.2-72.8) | 45%  (20; 23.1-68.5) | 40%  (20; 19.1-63.9) |  | 0%  (21; 0-16.1) | 0%  (20; 0-16.8) | 0%  (20; 0-16.8) | 0%  (20; 0-16.8) | 0%  (20; 0-16.8) |
|  |  | 20°C Var | 0%  (19; 0-17.6) | 9.52%  (21; 1.2-30.4) | 40%  (20; 19.1-63.9) | 70.58%  (17; 44.0-89.7) | 70%  (20; 45.7-88.1) |  | 0%  (17; 0-19.5) | 0%  (10; 0-30.8) | 0%  (18; 0-18.5) | 0%  (20; 0-16.8) | 0%  (20; 0-16.8) |
|  |  | 28°C | 10.52%  (19; 1.3-33.1) | 72.22%  (18; 46.5-90.3) | 47.36%  (19; 24.4-71.1) | 40%  (20; 19.1-63.9) | 15%  (20; 3.2-37.9) |  | 0%  (19; 0-17.6) | 5%  (20; 0.1-24.9) | 0%  (21; 0-16.1) | 0%  (19; 0-17.6) | 42.10%  (19; 23.1-68.5) |
|  | | | | | | | | | | | | | |
| **1209m** | DE | 20°C | 18.18%  (11; 2.3-51.8) | 80%  (20; 56.3-94.3) | 93.33%  (15; 68.1-99.8) | 85%  (20; 62.1-96.8) | 95%  (20; 75.1-99.9) |  | 0%  (5; 0-52.2) | 0%  (10; 0-30.8) | 0%  (8; 0-36.9) | 0%  (10; 0-30.8) | 6.25%  (16; 0.2-30.2) |
|  |  | 20°C Var | 6.66%  (15; 0.2-31.9) | 61.11%  (18; 35.7-82.7) | 83.33%  (18; 58.6-96.4) | 89.47%  (19; 66.9-98.7) | 85%  (20; 62.1-96.8) |  | 0%  (10; 0-30.8) | 0%  (17; 0-19.5) | 0%  (19; 0-17.6) | 0%  (19; 0-17.6) | 0%  (19; 0-17.6) |
|  |  | 28°C | 63.63%  (11; 30.8-89.1) | 84.61%  (13; 54.6-98.1) | 91.66%  (12; 61.5-99.8) | 80%  (15; 51.9-95.7) | 95%  (20; 75.1-99.9) |  | 0%  (10; 0-30.8) | 30.76%  (13; 9.1-61.4) | 60%  (15; 32.3-83.7) | 53.84%  (13; 25.1-80.8) | 65%  (20; 40.8-84.6) |
|  |  |  |  |  |  |  |  |  |  |  |  |  |  |
|  | TE | 20°C | 0%  (11; 0-28.5) | 35%  (20; 15.4-59.2) | 86.67%  (15; 59.5-98.3) | 65%  (20; 40.8-84.6) | 55%  (20; 31.5-76.9) |  | 0%  (5; 0-52.2) | 0%  (10; 0-30.8) | 0%  (8; 0-36.9) | 0%  (10; 0-30.8) | 0%  (16; 0-20.6) |
|  |  | 20°C Var | 0%  (15; 0-21.8) | 16.66%  (18; 3.6-41.4) | 66.66%  (18; 41.0-86.7) | 63.15%  (19; 38.4-83.7) | 70%  (20; 45.7-88.1) |  | 0%  (10; 0-30.8) | 0%  (17; 0-19.5) | 0%  (19; 0-17.6) | 0%  (19; 0-17.6) | 0%  (19; 0-17.6) |
|  |  | 28°C | 9.09%  (11; 0.2-41.3) | 61.53%  (13; 31.6-86.1) | 33.33%  (12; 9.9-65.1) | 20%  (15; 4.3-48.1) | 30%  (20; 11.9-54.3) |  | 0%  (10; 0-30.8) | 0%  (13; 0-24.7) | 6.66%  (15; 0.2-31.9) | 0%  (13; 0-24.7) | 0%  (9; 0-16.8) |

DE, dissemination efficiency corresponding to the proportion of mosquitoes with infected head; TE, transmission efficiency referring to the proportion of mosquitoes with infectious saliva. In brackets, the number of mosquitoes examined and 95% confidence interval of the proportion (exact Binomial method).

**Table S5. Transmission rates (%) of four *Ae. albopictus* populations from Albania (149m, 542m, 762m, 1209m) infected with CHIKV and DENV, then incubated at three differe**nt temperatures (20°C, 20°C variable, and 28°C) and examined at five days post-infection (3, 7, 10, 14, and 21).

| **Population** |  | **CHIKV** | | | | |  | **DENV** | | | | |
| --- | --- | --- | --- | --- | --- | --- | --- | --- | --- | --- | --- | --- |
|  | Days post-infection | 3 | 7 | 10 | 14 | 21 |  | 3 | 7 | 10 | 14 | 21 |
| **149m** | 20°C | NA | 22.2%  (9; 2.8-60.0) | 88.9%  (9; 51.8-99.7) | 72.7%  (11; 39-94) | 81.8%  (11; 48.2-97.7) |  | NA | NA | NA | 0%  (1; 0-97.5) | 0%  (1; 0-97.5) |
|  | 20°C Variable | NA | 37.5%  (8; 8.5-75.5) | 70%  (10; 34.8-93.3) | 62.5%  (16; 35.4-84.8) | 69.2%  (13; 38.6-90.9) |  | NA | NA | NA | NA | NA |
|  | 28°C | 42.9%  (7; 9.9-81.6) | 80.0%  (15; 51.9-95.7) | 50%  (10; 18.7-81.3) | 57.1%  (14; 28.9-82.3) | 36.4%  (11; 10.9-69.2) |  | NA | 0%  (1; 0-97.5) | 0%  (9; 0-33.6) | 12.5%  (16; 1.6-38.3) | 29.4%  (17; 10.3-56.0) |
|  | | | | | | | | | | | | |
| **542m** | 20°C | 100%  (1; 2.5-100) | 42.9%  (7; 9.9-81.6) | 81.8%  (11; 48.2-97.7) | 66.7%  (9; 29.9-92.5) | 66.7%  (18; 41-86.7) |  | NA | NA | NA | NA | NA |
|  | 20°C Variable | 33.3%  (3; 0.8-90.6) | 28.6%  (7; 3.7-71) | 88.9%  (9; 51.8-99.7) | 76.5%  (17; 50.1-93.2) | 53.8%  (13; 25.1-80.8) |  | NA | NA | NA | NA | NA |
|  | 28°C | 25%  (4; 0.6-80.6) | 70%  (10; 34.8-93.3) | 50%  (10; 18.7-81.3) | 30.8%  (13; 9.1-61.4) | 27.8%  (18; 9.7-53.5) |  | NA | 0%  (1; 0-97.5) | 33.3%  (3; 0.8-90.6) | 14.3%  (7; 0.4-57.9) | 18.8%  (16; 4.0-45.6) |
|  | | | | | | | | | | | | |
| **762m** | 20°C | NA | 20%  (10; 2.5-55.6) | 76.9%  (13; 46.2-95) | 56.2%  (16; 29.9-80.2) | 42.1%  (19; 20.3-66.5) |  | NA | NA | NA | NA | 0%  (1; 0-97.5) |
|  | 20°C Variable | 0%  (1; 0-97.5) | 20%  (10; 2.5-55.6) | 47.1%  (17; 23-72.2) | 75%  (16; 47.6-92.7) | 70%  (20; 45.7-88.1) |  | NA | NA | NA | NA | NA |
|  | 28°C | 18.2%  (11; 2.3-51.8) | 81.2%  (16; 54.4-96) | 50%  (18; 26-74) | 47.1%  (17; 23-72.2) | 15.8%  (19; 3.4-39.6) |  | NA | 33.3%  (3; 0.8-90.6) | 0%  (5; 0-52.2) | 0%  (13; 0-24.7) | 47.4%  (19; 24.45-71.1) |
|  | | | | | | | | | | | | |
| **1209m** | 20°C | 0%  (2; 0-84.2) | 43.8%  (16; 19.8-70.1) | 92.9%  (14; 66.1-99.8) | 76.5%  (17; 50.1-93.2) | 57.9%  (19; 33.5-79.7) |  | NA | NA | NA | NA | 0%  (1; 0-97.5) |
|  | 20°C Variable | 0%  (1; 0-97.5) | 27.3%  (11; 6-61) | 80%  (15; 51.9-95.7) | 70.6%  (17; 44-89.7) | 82.4%  (17; 56.5-96.2) |  | NA | NA | NA | NA | NA |
|  | 28°C | 14.3%  (7; 0.4-57.9) | 72.7%  (11; 39-94) | 36.4%  (11; 10.9-69.2) | 25%  (12; 5.5-57.2) | 31.6%  (19; 12.67.99-56.6) |  | NA | 0%  (4; 0-60.2) | 11.1%  (9; 0.3-48.2) | 0%  (7; 0-41) | 0%  (13; 0-24.7) |

In brackets, the number of mosquitoes examined and 95% confidence interval of the proportion (exact Binomial method). In case no moquitoes were able to disseminate the virus, cells are filled with NA.

**Table S6. Mean number of viral particles (± SD) detected in saliva of *Ae. albopictus* populations from Albania (149m, 542m, 762m, 1209m) infected with CHIKV and DENV, then incubated at 3 different incubation temperatures (20°C, 20°C variable, and 28°C) and examined at 5 days post-infection (3, 7, 10, 14, and 21).**

| Population | Virus | **CHIKV** | | | | |  | **DENV** | | | | |
| --- | --- | --- | --- | --- | --- | --- | --- | --- | --- | --- | --- | --- |
|  | Days post-infection | 3 | 7 | 10 | 14 | 21 |  | 3 | 7 | 10 | 14 | 21 |
| **149m** | 20°C | - | 38±45  (2) | 34±67  (8) | 10±8  (8) | 21±28  (9) |  | - | - | - | - | - |
|  | 20°C Variable | - | 35±22  (3) | 27±28  (7) | 59±121  (10) | 103±199  (9) |  | - | - | - | - | - |
|  | 28°C | 5±4  (3) | 58±121  (12) | 16±22  (5) | 6±3  (8) | 4±2  (4) |  | - | - | - | 17±16  (2) | 23±21  (5) |
|  | | | | | | | | | | | | |
| **542m** | 20°C | 24  (1) | 40±52  (3) | 374±987  (9) | 22±15  (6) | 25±44  (12) |  | - | - | - | - | - |
|  | 20°C Variable | 4  (1) | 55±63  (2) | 85±106  (8) | 53±70  (13) | 16±13  (7) |  | - | - | - | - | - |
|  | 28°C | 18  (1) | 11±13  (7) | 31±36  (5) | 38±70  (4) | 20±12  (5) |  | - | - | 4  (1) | 2  (1) | 42±66  (3) |
|  | | | | | | | | | | | | |
| **762m** | 20°C | - | 31±13  (2) | 104±246  (10) | 140±266  (9) | 554±986  (8) |  | - | - | - | - | - |
|  | 20°C Variable | - | 8±3  (2) | 6±8  (8) | 32±56  (12) | 98±261  (14) |  | - | - | - | - | - |
|  | 28°C | 1504±2116  (2) | 414±825  (13) | 39±34  (9) | 37±33  (8) | 13±16  (3) |  | - | 2  (1) | - | - | 19±33  (8) |
|  | | | | | | | | | | | | |
| **1209m** | 20°C | - | 23±23  (7) | 29±28  (13) | 223±567  (13) | 32±89  (11) |  | - | - | - | - | - |
|  | 20°C Variable | - | 29±22  (3) | 30±35  (12) | 170±514  (12) | 54±103  (14) |  | - | - | - | - | - |
|  | 28°C | 100  (1) | 24±25  (8) | 11±8  (4) | 105±169  (3) | 7±5  (6) |  | - | - | 110  (1) | - | - |

In brackets, the number of mosquitoes examined.
